# Evaluation of Cytotoxicity, Cell Attachment, and Elemental Characterization of Three Calcium Silicate-Based Sealers

**DOI:** 10.3390/ma16206705

**Published:** 2023-10-16

**Authors:** Anahi de Paula Melo, Camila Maria Peres de Rosatto, Danilo Cassiano Ferraz, Gabriela Leite de Souza, Camilla Christian Gomes Moura

**Affiliations:** Department of Endodontics, School of Dentistry, Federal University of Uberlândia, Uberlândia 38405-318, MG, Brazil; anahi.melo@ufu.br (A.d.P.M.); camilamprosattod@gmail.com (C.M.P.d.R.); daniloferraz@ufu.br (D.C.F.); gabrielaleiteodon@gmail.com (G.L.d.S.)

**Keywords:** calcium silicate, cytotoxicity, in vitro model, physicochemical properties, root canal filling materials

## Abstract

We investigated three calcium silicate-based sealers with respect to their chemical characterization, cytotoxicity, and attachment to RAW264.7 cells. BioRoot RCS (BR), Bio-C Sealer (BC), and Sealer Plus BC (SPBC) were assessed using Fourier transform infrared spectroscopy (FTIR), X-ray fluorescence spectroscopy (XRF), and energy-dispersive X-ray spectroscopy (EDX) (n = 4) for elemental characterization, and using scanning electron microscopy (SEM) to evaluate cell morphology and adhesion. Cytotoxicity was determined at different dilutions (1:1, 1:2, and 1:5) using the succinate dehydrogenase activity (MTT assay). Statistical analysis was performed for normal distribution using the Shapiro–Wilk test and for homoscedasticity using Levene’s test, and one-way ANOVA, Tukey’s/Dunnett’s post hoc tests for cell viability and XRF (α = 0.05). Calcium silicate hydrate and calcium hydroxide were detected by FTIR in all groups. EDX detected a higher calcium content for BR and SPBC and aluminum only in the premixed sealers. XRF detected the highest calcium release in BR (*p* < 0.05). The surface morphology showed irregular precipitates for all the sealers. SPBC at a 1:2 dilution resulted in the lowest cell viability compared to BR (*p* < 0.05) and BC (*p* < 0.05). The calcium silicate-based sealers produced a statistically significant reduction in cellular viability at a 1:1 dilution compared to the control group (*p* < 0.0001). All the sealers maintained viability above 70%.

## 1. Introduction

Verifying the properties of endodontic cements is extremely important to predict the clinical behavior of the material [1,2,3]. The biocompatibility of calcium silicate-based cements (CSBSs) is a crucial property [4], since these materials are placed in contact with the periapical tissues [3]. They are hydraulic cements composed mainly of tricalcium silicate, dicalcium silicate, and tricalcium aluminate, among other mineral oxides [5]. The intrinsic capacity of these materials to promote repair in their interaction with periapical tissues is attributed to their high alkalinity, which favors repair by mineralized tissue [6,7]. Central to this repair mechanism is the hydration process during cement setting, producing calcium hydroxide that releases essential hydroxyl ions upon dissociation [8]. The extent of biological responses observed in CSBSs, as evidenced by in vitro and in vivo studies [9,10], appears to depend on variations in the composition and proportions of compounds within these materials [11].

Delving deeper into the material’s composition and surface structure is extremely important in discerning its bioactivity [12]. Differences in biomaterial composition can imply variations in ionic interactions with surrounding tissues and cellular responses [13,14]. Macrophages provide an enlightening experimental model to evaluate the impact of sealants on cells involved in immunoinflammatory events in the periapical region [15].

BioRoot RCS (BR) (Septodont, Saint-Maur-des-Fossés, France) is a cement presented in powder–liquid form. It has adequate physicochemical properties, is biocompatible, and does not induce pro-inflammatory cytokines [16,17]. Bio-C Sealer (BC) (Angelus, Londrina, PR, Brazil) and Sealer Plus BC (SPBC) (MK Life, Porto Alegre, RS, Brazil) are ready-to-use CSBSs. Given the demonstrated in vivo biocompatibility of these materials [9], their behavior in macrophage cell cultures is a crucial aspect that remains to be clarified. Although physicochemical tests have helped to characterize the material [18,19], there is a lack of research that investigates elemental behavior through complementary techniques. No previous studies have evaluated the elemental composition of Sealer Plus BC.

This current study strives to not only evaluate the elemental composition, but also to compare the biological attributes of three CSBSs (BR, BC, and SPBC) within the RAW264.7 cell line, with respect to cellular cytotoxicity, attachment, compound leaching, and surface morphology. The null hypothesis was that the tested sealants would not present differences in chemical properties and biological effects.

## 2. Materials and Methods

### 2.1. Sample Preparation

The sealers were placed into sterile silicone molds (7.75 mm in diameter × 1.5 mm in thickness) and placed in a humidified chamber at 37 °C for 24 h to facilitate setting [18]. The proportions and specifications are detailed in Table 1. To ensure uniform conditions across all the groups, BR was inserted into the syringes immediately prior deposition into the molds.

Upon completion of the setting phase, sealer discs (n = 4) were extracted from the molds and placed within sterile plastic tubes containing 10 mL of minimum essential medium Eagle (α-MEM) (LGC Biotecnologia, Cotia, SP, Brazil), supplemented with 1% penicillin–streptomycin (Sigma-Aldrich, Saint Louis, MO, United States). After a 24 h incubation period, the medium for each sealer specimen was carefully transferred to a sample cup (model SC-4340, PremierLab Supply, Port St. Lucie, FL, United States) for X-ray fluorescence (XRF) analysis (model S8 Tiger Series 2, Bruker, Kontich, ANR, Belgium). Concurrently, the corresponding sealer disc underwent preparation for scanning electron microscope (SEM) and energy dispersive X-ray (EDX) analysis.

### 2.2. Elemental Composition Analysis

A quantitative assessment of the released components from the sealers into the α-MEM medium was conducted using an XRF assay (S8 Tiger, Bruker, Karlsruhe, Germany). The specimens were measured using the Quant-Express standard method. SpectraPlus software v1.1.0.10 was employed to compute the concentrations of specific elements, including calcium (Ca), silicon (Si), and zirconium (Zr). This computation was based on the intensities of the X-ray peaks corresponding to each respective element.

### 2.3. SEM/EDX Characterization

Sealer discs (n = 4) were rinsed post-setting in 0.05 M Na-cacodylate buffer and then fixed in 2.5% glutaraldehyde within 0.1 M Na-cacodylate buffer. Following this, a dehydration process using a series of graded ethanol solutions (ranging from 50% to 100%) was carried out, culminating in hexamethyldisilazane-assisted drying. Subsequently, the dried specimens were affixed onto stubs and coated with a 30 nm layer of gold using sputter-coating (Leica EM SCD050 tabletop device, Wetzlar, HE, Germany).

For qualitative evaluation, SEM (EVO 10, Carl Zeiss, Oberkochen, BW, Germany) analysis was performed across three distinct regions at magnifications of 700×, 2340×, and 5000×. It was stablished an accelerating voltage of 5.0 kV with a 20 µm aperture. EDX (Oxford model 51-ADD0048, Abingdon, OXON, United Kingdom) analysis was conducted at a working distance of 8.5 mm, and each point of interest underwent a 30 s acquisition time.

### 2.4. FTIR Characterization

Fourier transform infrared (FTIR) spectroscopy (Vertex 70, Bruker Optik GmbH, Ettlingen, BW, Germany) was employed to evaluate the absorbance bands corresponding to distinct functional groups within the CSBSs. The analysis was conducted using the OPUS v.7.8; Bruker Optik software program. The sealer discs (n = 4) were prepared similarly to the SEM/EDX and XRF analyses. The specimens were positioned within the diamond device window, and the holder’s head was adjusted to establish contact with the material surface. Spectra were obtained across the range of 4000–400 cm^−1^, employing a resolution of 4 cm^−1^ over 32 scans using transmittance spectroscopy.

### 2.5. Cytotoxicity

Fresh CSBSs (0.22 mL) were inserted into 24-well plates under aseptic conditions in a laminar flow cabinet. All the materials were promptly overlaid with 2.5 mL of α-MEM containing 1% penicillin–streptomycin (Sigma-Aldrich), supplemented with 10% heat-inactivated fetal bovine serum (FBS), and further enriched with 10% α-MEM (LGC Biotecnologia, Cotia, SP, Brazil). Following this, an incubation period of 24 h at 37 °C was employed.

The original extracts (1:1) were prepared following ISO 10993-5/2009 recommendations [20]. The medium was collected, filtered through 0.22 µm filters, and diluted to concentrations of 1:1, 1:2, and 1:5 [21]. RAW264.7 cells were seeded in 96-well culture plates (1 × 10^4^ cells/mL) in 10% α-MEM. Following cell adherence, the effect of the sealer extracts (1:1, 1:2, and 1:5 dilutions) on cell viability at 24 h was assessed using the MTT assay, as was previously described [22]. The control group of cells remained unexposed to any sealer extracts. Each dilution was subjected to analysis in triplicate in three independent experiments by a single experienced operator. Data from each group were analyzed by an independent operator and presented as mean ± standard deviation.

### 2.6. Cell Attachment

To assess cell attachment, a direct testing approach was employed. RAW264.7 cells (passages 5–7; BCBRJ, Rio de Janeiro, RJ, Brazil) were seeded at a density of 2 × 10^4^ cells on the surface of the sealer discs. This seeding took place within 48-well culture plates containing 10% α-MEM and spanned a 24 h period (n = 4). Subsequently, the specimens underwent the same processing and SEM analysis as previously outlined. As a comparative control, cells were also seeded on glass coverslips.

### 2.7. Statistical Analysis

The data were analyzed for normal distribution using the Shapiro–Wilk test and for homoscedasticity using the Levene test. One-way ANOVA complemented by Tukey’s post hoc test was used to perform XRF analysis. Cell viability was analyzed by comparing different extract dilutions of the sealer. Next, the sealers were compared with one another at the same dilution and later with the controls using one-way ANOVA, Tukey’s test, and Dunnett’s test. This study was mostly qualitative. The level of significance was set at *p* < 0.05, and all analyses were performed using GraphPad Prism software program v8.2.0 (San Diego, CA, United States).

## 3. Results

### 3.1. XRF Analysis

Table 2 provides the leaching results of elements from hydrated sealers, expressed in parts per million (ppm). BR exhibited high release of calcium ions into the solution at 24 h, differing significantly from SPBC and BC (*p* < 0.05). However, all the sealers exhibited a low level of silicon leaching, with no significant differences among the groups (*p* < 0.05). Zirconium was detected only in the BC samples (*p* < 0.05).

### 3.2. SEM-EDX Analysis

**Figure 1:**
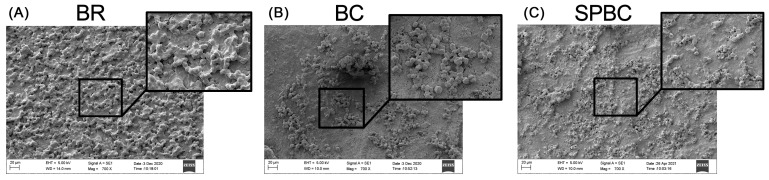
Scanning electron microscopy (SEM) of hydrated CSBS surface morphology at 700× and 2340× magnification after 24 h in α-MEM: BR (**A**), BC (**B**), and SPBC (**C**).

**Figure 2:**
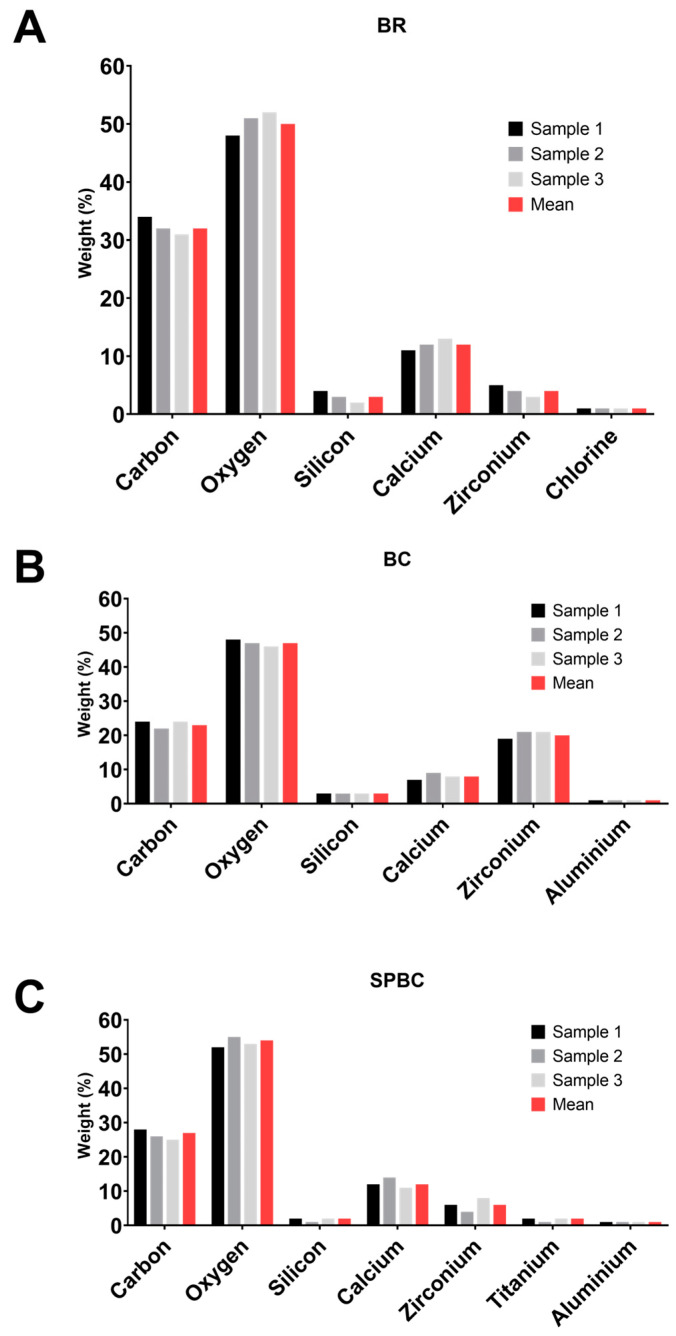
Energy dispersive X-ray (EDX) results of the CSBS chemical elements: BR (**A**), BC (**B**), and SPBC (**C**).

Figure 1 displays representative SEM images of the hydrated sealer discs. The corresponding EDX analysis is shown in Figure 2. The surface morphology of all the sealers showed distinct crystallite deposits and crystal precipitates, each exhibiting a unique crystalline structure. BR exhibited a suggestive globular and cubical crystallite appearance, with a mostly grainy microstructure (Figure 1A) and more numerous deposits of crystals compared to BC and SPBC. BC had the most regular surface (Figure 1B), whereas SPBC’s surface particles exhibited varied morphologies, notably coarse and elongated structures (Figure 1C).

EDX analysis highlighted the presence of carbon, calcium (Ca), zirconium (Zr), silicon (Si), and oxygen peaks for all the CSBSs (Figure 2A–C). Of the sealers, BR and SPBC showcased higher Ca content, BC exhibited notable Zr content, and all displayed low Si content, particularly SPBC. The main difference in composition was the presence of aluminum (Al) in BC and SPBC, titanium (Ti) in SPBC, and chlorine (Cl) in BR.

### 3.3. FTIR Analysis

**Figure 3:**
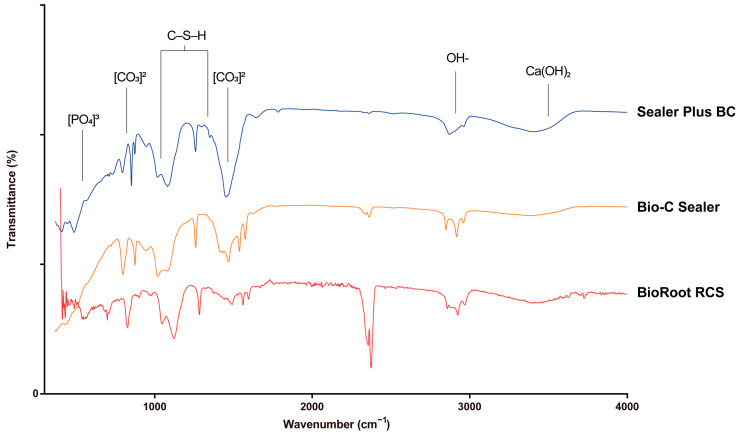
Illustrations of Fourier transform infrared spectroscopy (FTIR) for CSBSs: BR, BC, SPBC.

Table 3 and Figure 3 depict detailed FTIR absorbance spectra of the CSBSs. A qualitative comparison of FTIR spectra revealed slight differences for each sealer in relation to the spectroscopic plots and intensities. Ion phosphate was identified in all groups at 560 and 600 cm^–1^ [23]. Characteristic bands indicating the formation of calcium silicate hydrate (CSH) were present in all the sealers, recognizable from ~900 to ~1200 cm^–1^ and at 1350 cm^–1^, signifying Si-O combinations [23,24]. A minor absorption band around ~3640 cm^–1^ indicated calcium hydroxide formation [25,26,27], particularly evident in SPBC. The spectrum also indicates the absorption intensities of carbonates at 878 and ~1400 cm^–1^ [23,24,28].

### 3.4. Cell Morphology, Adhesion and Viability

**Figure 4:**
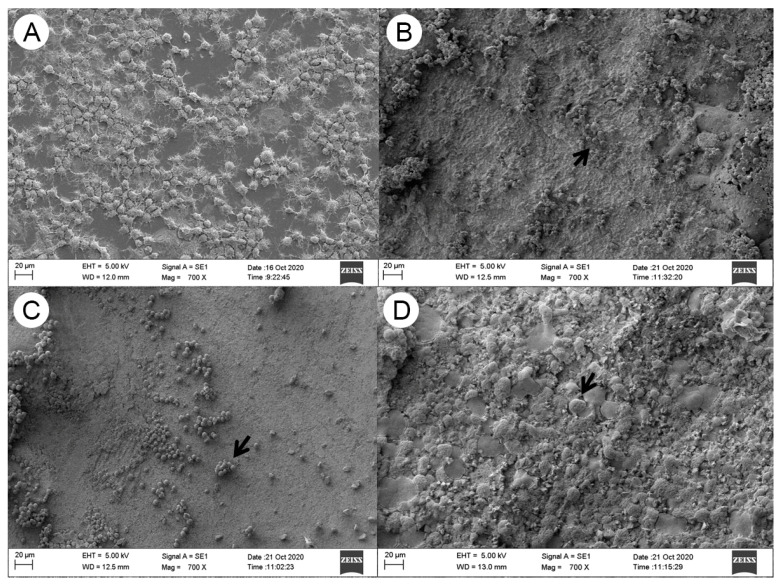
Cell attachment of RAW264.7 onto the CSBS surfaces (Control: (**A**); BR: (**B**); BC: (**C**); SPBC: (**D**)). The arrows indicate areas with cells.

Qualitative examination of the RAW264.7 cells revealed mainly round cells and debris on the sealer surfaces, indicating cell death. The control groups exhibited better adhesion and spread on the coverslips (Figure 4).

**Figure 5:**
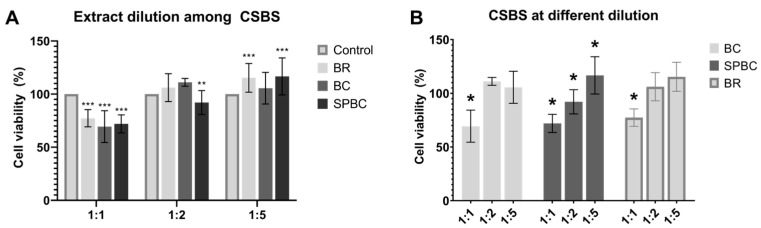
The extract dilution effects of CSBSs on cell viability of RAW264.7 evaluated using the MTT formazan assay. We considered 100% cell viability for the control group: (**A**) Asterisks allow comparisons among different extract dilution for the same sealer; (**B**) comparisons among CSBSs and with the control group. * *p* < 0.05 compared intragroup; ** *p* < 0.05 compared intergroup at same extract dilution; *** *p* < 0.05 compared with the controls.

Cell viability assessment revealed intragroup differences in BR and BC at different dilutions, with lower viable cell percentages at 1:1 dilution (*p* < 0.0001). SPBC exhibited variations across all dilutions (*p* < 0.0001), showing higher viability at 1:5 dilution (Figure 5A). Intergroup analysis displayed no significant differences between the sealers at 1:1 and 1:5 dilutions (*p* > 0.0001), while at 1:2 dilution, SPBC exhibited lower cell viability compared to BR and BC (*p* < 0.0001) (Figure 5B). Comparatively, the CSBSs at 1:1 dilution significantly reduced cellular viability compared to the control group (*p* < 0.0001). However, BR and SPBC exhibited higher viability at 1:5 dilution compared to the controls (*p* < 0.0001) (Figure 5B).

## 4. Discussion

The null hypothesis was rejected as the CSBSs demonstrated significant differences across several aspects. This study marks the first attempt to comprehensively compare the elemental behavior of SPBC using a combination of XRF, FTIR, and SEM-EDX methodologies.

FTIR and SEM-EDX have already been used to elucidate the formation of calcium phosphate and hydroxyapatite deposits on CSBSs after soaking them in different solutions [29,30]. When exposed to humidity, CSBSs incorporate water to initiate the setting reaction, yielding C–S–H gel [20], which subsequently reacts with the sealer to produce calcium hydroxide [31]. Slight variations in chemical composition may occur due to equipment and measurement differences in EDX analyses. This study relied on the average of three measurements for each sample to avoid biases in determining composition content. In the present study were observed bands (~3640 cm^−1^) associated with calcium hydroxide formation, consistent with prior research [25]. Moisture in these endodontic sealers facilitates the creation of a suitable surface for calcium phosphate and apatite layer formation, both of which contribute to the induction of hard tissue deposition [1]. BR showed greater calcium release in 24 h when compared to BC and SPBC. Differences in ion release may influence the clinical sealing capacity or the characteristics of the mineral bond formed to the dentin substrate [32]. Our findings align with previous FTIR analyses of BR and BC, reaffirming the presence of phosphate ions [23,26,33].

Even though similar structures were identified in the FTIR spectrum, the bands of BR varied qualitatively compared to those of premixed sealers. This difference may be attributed to BR unique status as a two-component sealer evaluated [33], influenced by potential powder–liquid ratio alterations during mixing [33]. Moreover, the organic structures inherent in premixed sealers, acting as thickeners or fillers, set them apart from BR [33]. The prolonged setting reaction, particularly prevalent in premixed materials [33], could have contributed to the observed decrease in the peak intensity of FTIR-detected functional groups.

XRF analysis was employed to monitor ion release from the CSBSs into α-MEM solution. In a clinical situation, fluids from apical tissues have the potential to infiltrate the root canal, triggering the release of ions from the sealers [34], a phenomenon exemplified in vitro by BR within this study. Although the biomineralization potential of these sealers in contact with apical tissues was not evaluated in this study, all the sealers demonstrated calcium ion release. BR displayed the highest calcium content, consistent with prior study [35]. Additionally, higher solubility values compared to ISO 6876:2012 [36] recommendations have been reported for BR, potentially indicating heightened calcium ion dissolution rates [37]. Although the solubility rates of CSBSs are still controversial, this may indicate the high dissolution rates of calcium ions for BR presented in this study. Zirconium content was found in all the sealers tested, which provides suitable radiopacity and does not hinder the hydration of calcium silicate-based products [17]. Zirconium oxide offers better biocompatibility and enhanced physical and mechanical properties, and prevents tooth discoloration when compared to bismuth oxide [17,38].

Cellular evaluations have been previously conducted on the same sealers using alternate cell models [9,39]. However, the host response pattern for different biomaterials varied according to cell type [10]. Given the vital role of macrophages in inflammatory response and periapical area repair, their assessment in in vitro models for novel materials remains pertinent [40]. Regarding cell viability, the results showed a lower percentage of viable cells for SPBC. However, it is important to note that a direct comparison with a prior study is not feasible due to the distinct response of fibroblasts and the significantly higher dilutions used in the current investigation [41].

The direct interaction between RAW264.7 cells and the surfaces of the sealers provided insights into the impact on cell adhesion and morphology. While the exact mechanisms behind cell detachment remain uncertain, the higher number of cells on the control cover slips suggests that detachment could be due to the dissolution of the superficial sealer layer in the culture medium. RAW264.7 cells are a well-known and widely used immortalized cell line for studies in endodontics [42,43].

## 5. Conclusions

Among the different CSBSs evaluated in this study, it was observed that BR demonstrated the highest calcium content. In contrast, the premixed sealers, BC and SPBC, displayed similar compositions characterized by the presence of heavy metals like aluminum. All the sealers maintained viability exceeding 70%. This finding underscores the potential feasibility of these sealers for practical applications in a clinical setting.

## Figures and Tables

**Table 1:** Tested materials, followed by manufacturer, composition, and proportion.

Groups	Materials	Manufacturer	Composition	Proportion
BR	BioRoot RCS	Septodont, Saint-Maur-des-Fossés, France	Powder: tricalcium silicate, zirconium oxide and povidone Liquid: aqueous solution of calcium chloride and polycarboxylate	1 spoon of powder + 6 drops of liquid
BC	Bio-C Sealer	Angelus, Londrina, PR, Brazil	Tricalcium silicate, dicalcium silicate, tricalcium aluminate, calcium oxide, zirconium oxide, silicon oxide, polyethylene glycol, iron oxide	Ready to use
SPBC	Sealer Plus BC	MK Life, Porto Alegre, RS, Brazil	Calcium disilicate, calcium trisilicate, zirconium oxide, calcium hydroxide, propylene glycol	Ready to use

**Table 2:** Elements of hydrated CSBSs leached in α-MEM solution according to XRF analysis (ppm).

	CSBS			
Elements Identified	BR	BC	SPBC	*p*
Calcium (Ca)	3200	200	800	0.0201
Silicon (Si)	19	20	12	0.5302
Zirconium (Zr)	NI	4	NI	0.4219

CSBS, calcium silicate-based sealer; BR, BioRoot RCS; BC, Bio-C Sealer; SPBC, Sealer Plus BC; NI, not identified. *p* value indicates statistical difference among calcium silicate-based sealers (*p* < 0.05).

**Table 3:** Representation of peaks of interest and components with respective wavenumber (cm^−1^) detected by FTIR analysis for each tested sealer.

Wavenumber (cm^−1^)	BioRoot RCS	Bio-C Sealer	Sealer Plus BC Sealer	Reference
878	[CO_3_]^2−^	[CO_3_]^2^	[CO_3_]^2^	[23,24,28]
~1400	[CO_3_]^2^	[CO_3_]^2^	[CO_3_]^2^	[23,24,28]
~900–1200	C–S–H	C–S–H	C–S–H	[23,24]
1350	C–S–H	C–S–H	C–S–H	[23,24]
~3640	Ca(OH)_2_	Ca(OH)_2_	* Ca(OH)_2_	[25,26,27]
560	[PO_4_]^3−^	[PO_4_]^3−^	[PO_4_]^3−^	[23]
600	[PO_4_]^3−^	[PO_4_]^3−^	[PO_4_]^3−^	[23]

[CO_3_]^2−^, carbonate; C–S–H, calcium silicate hydrate; Ca(OH)_2_, calcium hydroxide; * Ca(OH)_2_, peak more evident in SPBC; [PO_4_]^3−^, phosphate ions.

## Data Availability

No new data were created or analyzed in this study. Data sharing is not applicable to this article.
